# Changes in the Mean of Medical Visits Due to Psychiatric Disease in Korean Children and Adolescents before and during the COVID-19 Pandemic

**DOI:** 10.3390/life12040600

**Published:** 2022-04-18

**Authors:** So Young Kim, Na-Eun Lee, Dae Myoung Yoo, Ji Hee Kim, Mi Jung Kwon, Joo-Hee Kim, Woo Jin Bang, Hyo Geun Choi

**Affiliations:** 1 Department of Otorhinolaryngology-Head & Neck Surgery, CHA Bundang Medical Center, CHA University, Seongnam 13496, Korea; sossi81@hanmail.net; 2 Hallym Data Science Laboratory, Hallym University College of Medicine, Anyang 14068, Korea; nel2001@hanmail.net (N.-E.L.); ydm1285@naver.com (D.M.Y.); 3 Department of Neurosurgery, Hallym University College of Medicine, Anyang 14068, Korea; kimjihee.ns@gmail.com; 4 Department of Pathology, Hallym University College of Medicine, Anyang 14068, Korea; mulank@hanmail.net; 5 Division of Pulmonary, Allergy, and Critical Care Medicine, Department of Medicine, Hallym University College of Medicine, Anyang 14068, Korea; luxjhee@gmail.com; 6 Department of Urology, Hallym University College of Medicine, Anyang 14068, Korea; yybang@gmail.com; 7 Department of Otorhinolaryngology-Head & Neck Surgery, Hallym University College of Medicine, Anyang 14068, Korea

**Keywords:** COVID-19, depression, anxiety, child, adolescent, epidemiology

## Abstract

The COVID-19 pandemic has been suggested to have adverse impacts on psychiatric disorders. This study aimed to investigate the changes in medical visits due to a wide range of psychiatric disorders in children during the COVID-19 pandemic. The medical visits of all Korean children and adolescents (0–19 years old) due to the 12 following psychiatric disorders were investigated: autism; attention-deficit/hyperactivity disorder (ADHD); depressive disorder; bipolar disorder; primary insomnia; schizophrenia; panic disorder; hypochondriasis; posttraumatic stress disorder (PTSD); anxiety disorder; anorexia nervosa; and adephagia. The mean medical visits before and during the COVID-19 pandemic were compared. The mean number of clinical visits due to autism, ADHD, depressive disorder, bipolar disorder, panic disorder, hypochondriasis, PTSD, anxiety disorder, and anorexia nervosa was higher during the COVID-19 pandemic than before the COVID-19 pandemic (all *p* < 0.05). The higher mean number of medical visits due to psychiatric disorders was maintained in age and sex subgroups. The female and adolescent groups demonstrated a higher mean number of medical visits due to psychiatric disorders during the COVID-19 pandemic. The medical visits due to many psychiatric disorders were higher during the COVID-19 pandemic than before COVID-19 in children and adolescents in Korea. Women and adolescents were more susceptible to psychiatric disorders during the COVID-19 pandemic.

## 1. Introduction

The coronavirus disease 2019 (COVID-19) pandemic has been suggested to increase psychiatric burdens [1,2]. In particular, the COVID-19 pandemic could impose specific circumstances on children compared to adults. The closure of daycare centers or schools during the COVID-19 lockdown or social distancing acts may disrupt social support for susceptible children, such as those with low socioeconomic status, and increase the risk of abuse at home [3,4]. The widespread use of home-based online learning programs can increase the risk of internet addiction [5]. In addition, childhood is a period of development for both physical and psychiatric aspects and children are vulnerable to adverse or traumatic events [6,7]. Posttraumatic stress disorder (PTSD) in children is reported to be characterized by induced developmental abnormalities in the frontolimbic circuits, which result in increased threat reactivity and the loss of emotional control [7]. Children have an immature cognitive capacity and can experience hardships in coping with the COVID-19 pandemic [6]. Therefore, the psychiatric impacts of the COVID-19 pandemic on children need to be evaluated separately from the impacts on adults.

Several recent studies have reported the effect of COVID-19 on psychiatric wellbeing of children [8,9]. Our previous study estimated that self-reported stress and suicide-related behaviors were not increased in Korean adolescents during the COVID-19 pandemic [10]. However, this survey was based on self-report questionnaires, and the age range was restricted to the adolescent period (aged 12 to 18 years). Other prior studies have suggested increased anxiety and depression in children during the COVID-19 pandemic [8]. In a meta-analysis study, children and adolescents were exposed to an increased risk of anxiety, depression, sleep disturbances, and anorexia, which were related to containment actions, such as social distancing, school closures, and isolation [9]. Due to quarantine measures, social isolation and restrictions on physical activities can result in loneliness and mental distress. During the lockdown period, emotional and somatic symptoms, including depression, anxiety, uncontrolled emotional problems, and somatic complaints, increased in the 8- to 18-year-old population in Spain [11]. Because children are in developmental periods and have poorer coping skills for disasters than adults, various psychiatric disorders, in addition to depression and anxiety, could be influenced by the COVID-19 pandemic.

This study predicted that medical visits for a wide range of psychiatric disorders in youth would be changed during the COVID-19 pandemic compared to those before the COVID-19 pandemic. To test this hypothesis, all Korean children were investigated for mean medical visits due to psychiatric disorders during and before the COVID-19 pandemic.

## 2. Materials and Methods

### 2.1. Ethics

The analyses of the national health insurance data in Korea for this study were permitted by the Ethics Committee of Hallym University (2021-11-004). Written informed consent was not required in this study because the Ethics Committee permitted the exemption of the acquisition of written informed consent.

### 2.2. Participants and Measurements

This study included the entire Korean children and adolescent population (~8.7 million, 0–19 years old) without exception, as a single health insurance system mandatorily covers the whole country. Thus, we could gather the data of all Koreans, from primary clinics to tertiary hospitals. In this study, we analyzed Korea National Health Insurance Database medical claim code data from January 2018 through May 2021. The data were extracted for age, sex, and the diagnostic claim codes of psychiatric diseases. As the first COVID-19 cases in Korea were discovered on 20 January 2020, and disease prevention and control started in March 2020, we defined the period of ‘before COVID-19′ as until February 2020 and the period ‘during COVID-19′ from March 2020 onward.

We evaluated the monthly incidence of 12 psychiatric diseases that are common in primary clinics. The patients’ diseases were examined based on the histories of visiting clinics in person for each psychiatric disorder. The diagnosis of psychiatric diseases were based on the following ICD-10 codes: autism (F840, F841); ADHD (F900); depressive disorder (F32, F33); bipolar disorder (F31); primary insomnia (F510, G470); schizophrenia (F20, F21, F231, F232, F25); panic disorder (F400, F410); hypochondriasis (F452); posttraumatic stress disorder (F431); anxiety disorder (F40, F41); anorexia nervosa (F500, F501, F508); and bulimia nervosa (F502, F503, F504, F505). The diagnostic codes were registered by the physicians. The incidence of diseases was calculated without duplication, as we had the health insurance database of the entire hospitals or clinics, and patients were identified by their unique resident registration number.

### 2.3. Statistics

The differences in the mean medical visits for diseases before and during the COVID-19 pandemic were estimated using the Mann–Whitney U test for nonparametric values. The group difference in the variance in clinical visits due to psychiatric diseases before and during the COVID-19 pandemic was evaluated using Levene’s test for nonparametric values [12]. The participants were subgrouped according to age (0–4 years old, 5–9 years old, 10–14 years old, and 15–19 years old) and sex.

Two-tailed analyses were performed. Statistical significance was defined as *p* values < 0.05. SPSS version 22.0 was used for the statistical analyses (IBM, Armonk, NY, USA).

## 3. Results

**Figure 1:**
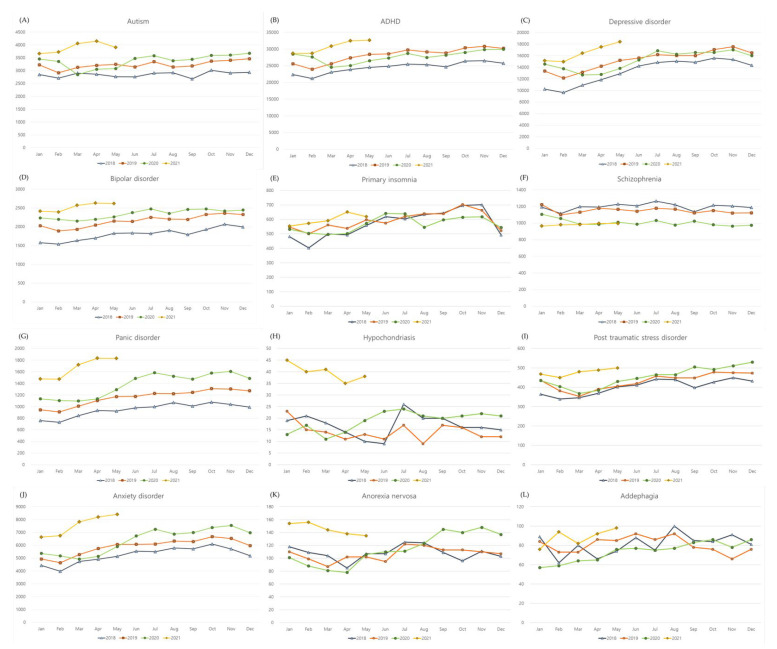
Monthly incidence of psychiatric diseases in 2018, 2019, 2020, and 2021.

The number of mean clinical visits due to autism, ADHD, depressive disorder, bipolar disorder, panic disorder, hypochondriasis, PTSD, anxiety disorder, and anorexia nervosa was higher during the COVID-19 pandemic than before the COVID-19 pandemic (all *p* < 0.05, Table 1 and Figure 1). On the other hand, the mean number of clinical visits due to schizophrenia was lower during the COVID-19 pandemic than before the COVID-19 pandemic (*p* < 0.001). The variance in clinical visits due to primary insomnia and schizophrenia was lower during the COVID-19 pandemic than before the COVID-19 (SD = 48.5 vs. 77.9, *p* < 0.001 for primary insomnia, SD = 19.7 vs. 48.9, *p* < 0.001 for schizophrenia).

According to sex, the mean number of clinical visits due to autism, depressive disorder, bipolar disorder, panic disorder, hypochondriasis, and anxiety disorder were higher in both the male and female groups during the COVID-19 pandemic than before the COVID-19 pandemic (all *p* < 0.05, Table 2). In the female group, the mean number of clinical visits due to ADHD, PTSD, and anorexia nervosa was higher during the COVID-19 pandemic than before the COVID-19 pandemic (all *p* < 0.05). The mean number of clinical visits due to schizophrenia was lower during the COVID-19 pandemic than before the COVID-19 pandemic in both sexes (all *p* < 0.001).

According to age, the 0–4-year-old group demonstrated higher mean number of clinical visits due to autism and ADHD during the COVID-19 pandemic than before the COVID-19 pandemic (all *p* < 0.05, Table 3). In the 5–9-year-old group, autism, depressive disorder, bipolar disorder, primary insomnia, anxiety disorder, and anorexia nervosa showed higher mean number of clinical visits during COVID-19 pandemic than before the COVID-19 pandemic (all *p* < 0.05). In the 10–14-year-old group, autism, ADHD, schizophrenia, panic disorder, hypochondriasis, anxiety disorder, and anorexia nervosa demonstrated higher mean number of clinical visits during the COVID-19 pandemic than before the COVID-19 pandemic (all *p* < 0.05). In the 15–19-year-old group, ADHD, depressive disorder, bipolar disorder, panic disorder, hypochondriasis, PTSD, anxiety disorder, and anorexia nervosa indicated higher mean number of clinical visits during the COVID-19 pandemic than before the COVID-19 pandemic (all *p* < 0.05). On the other hand, autism and schizophrenia showed lower mean number of clinical visits during the COVID-19 pandemic than before the COVID-19 pandemic (all *p* < 0.05). 

## 4. Discussion

Many psychiatric disorders, including autism, ADHD, depressive disorder, bipolar disorder, panic disorder, hypochondriasis, PTSD, anxiety disorder, and anorexia nervosa, demonstrated a higher mean number of clinical visits in children during the COVID-19 pandemic than before the COVID-19 pandemic. Females and adolescents demonstrated a greater mean number of medical visits due to psychiatric disorders during the COVID-19 pandemic than before the COVID-19 pandemic. On the other hand, the mean number of clinical visits due to schizophrenia was lower in children during the COVID-19 pandemic than before the COVID-19 pandemic.

A higher burden of psychiatric diseases in youth during the COVID-19 pandemic has been suggested in a number of recent studies [9,13,14,15]. In a cross-sectional online survey, the three most common psychiatric symptoms in primary and secondary school students during school closures due to COVID-19 outbreaks were anxiety, depression, and stress [13]. Another prior study reported that the psychiatric symptoms of inattention (30.8%) and sleep disturbances (21.3%) were also high in youth during the COVID-19 pandemic [14]. Especially, the children with pre-existing psychiatric diseases were suggested to show higher incidence of the psychiatric symptoms during COVID-19 pandemic in recent studies [16]. For instance, in children with ADHD, the psychiatric symptoms were higher during the COVID-19 pandemic [16]. A few potential risk factors can be postulated for the higher incidence of psychiatric symptoms during COVID-19 pandemic. It was suggested that school closures and the increased burden of family conflicts due to increased online education and home care could mediate the higher psychiatric symptoms in children during the COVID-19 pandemic [14]. Moreover, the confinement and developmental vulnerabilities of children and adolescents may have contributed to the increased number of medical visits due to psychiatric disorders during the COVID-19 pandemic in this study.

In addition to the common affective disorders of anxiety and depression, other psychiatric disorders of ADHD, panic disorder, hypochondriasis, and anorexia nervosa were increased during the COVID-19 pandemic in the present study. The increased time spent on media use is thought to aggravate the risk of ADHD in youth [17]. In an online survey, the abuse of smartphones and the internet was associated with severe depression in children during the COVID-19 pandemic [18]. Fear or panic due to the COVID-19 pandemic may influence the increased number of medical visits due to panic disorder and hypochondriasis in children. Quarantine strategies and health-related education programs could encourage attention to health and could evoke hypochondriasis. Furthermore, the immature coping skills and developing neurocognitive functions of children could make them vulnerable to the pandemic crisis and may increase psychiatric disorders.

On the other hand, the number of clinical visits due to schizophrenia decreased during the COVID-19 pandemic. The clinical features of schizophrenia, such as psychosis and cognitive deficits and low sociodemographic status, may impair medical care in patients with schizophrenia [19]. Moreover, patients with schizophrenia were reported to show a high risk of COVID-19 infection [20,21]. Thus, infection with COVID-19 could hinder proper treatment for patients with schizophrenia. Telepsychiatry applications were proposed to support the medical management of patients with schizophrenia [22].

According to age and sex, the female and adolescent groups demonstrated more psychiatric disorders with a higher number of clinical visits during the COVID-19 pandemic in this study. In line with the current results, a few previous studies have suggested a higher susceptibility of adolescents and females to anxiety and depression during the COVID-19 pandemic [8,23]. A cross-sectional online survey reported that adolescents had the highest number of emotional problems during the lockdown period among youth (1-19 years) [23]. The higher incidence of affective disorders during the adolescent period could contribute to the significant association of medical visits due to these psychiatric disorders with the COVID-19 pandemic.

This study investigated data from all Korean children for the diagnosis of psychiatric disorders. Because all Koreans are legally registered for the health insurance system with their respective resident registration numbers, there was little concern on the missing or overlapping data. However, a few shortcomings need to be addressed when generalizing the current results. First, the cohort population was composed of one ethnic group: Korean children. Thus, regional or ethnic differences can exist for the changes in the cases of psychiatric disorders and the COVID-19 pandemic [24]. In addition, stratified social distancing policies from the Korean government were enacted without complete lockdown events. Thus, there was no restriction to visit clinics during study periods. Second, undiagnosed and subclinical cases could have been missed in the current study. Because this study analyzed health claims data, patients who did not visit clinics could not be included. Third, the severity and management of psychiatric disorders were heterogeneous in the current study. Lastly, there may have potential confounders, such as eating disorders, the increase in time spent at home, the increase in family meals and supermarket, and the stigma on the search for psychiatric visits or on access to clinical and rehabilitation facilities during COVID-19 [25,26,27] To elucidate the long-term consequences of the effects of the COVID-19 pandemic on psychiatric disorders, a long-term follow-up study with detailed treatment histories may be warranted.

## 5. Conclusions

The diagnoses of autism, ADHD, depressive disorder, bipolar disorder, panic disorder, hypochondriasis, PTSD, anxiety disorder, and anorexia nervosa were increased in children during the COVID-19 pandemic. Females and older children were more susceptible to diagnoses of psychiatric disorders during the COVID-19 pandemic.

## Figures and Tables

**Table 1:** Mean, standard deviation of incidence of diseases before and during COVID-19, and their difference.

Diseases	Before COVID-19		During COVID-19		*p*-Values of Difference	
	Mean	SD	Mean	SD	Mean	Variance
Autism	3070.6	243.1	3550.6	359.1	<0.001 *	0.936
ADHD	26498.2	2600.5	28667.8	2332.6	0.021 *	0.480
Depressive disorder	14273.1	2036.9	15748.9	1656.6	0.021 *	0.856
Bipolar disorder	1996.0	237.7	2416.7	138.2	<0.001 *	0.140
Primary insomnia	575.5	77.9	583.9	48.5	0.745	0.037 †
Schizophrenia	1166.4	48.9	989.1	19.7	<0.001 *	0.014 †
Panic disorder	1058.0	158.5	1507.1	213.1	<0.001 *	0.159
Hypochondriasis	15.5	4.3	26.3	10.6	<0.001 *	0.796
Post traumatic stress disorder	416.2	40.1	465.5	45.1	0.001 *	0.766
Anxiety disorder	5536.6	675.1	6902.7	990.8	<0.001 *	0.739
Anorexia nervosa	106.4	10.8	127.1	24.8	0.003 *	0.106
Adephagia	79.2	10.9	80.6	9.6	0.725	0.496

* Mann–Whitney U test, significance at <0.05, † Levene’s test in non-parametric data, significance at <0.05.

**Table 2:** Mean, Standard deviation of incidence of diseases before and during COVID-19, and their difference in the subgroup by sex.

Diseases	Before COVID-19		During COVID-19		*p*-Values of Difference	
	Mean	SD	Mean	SD	Mean	Variance
Men						
Autism	2595.8	201.1	2960.7	288.3	<0.001 *	0.892
ADHD	21881.1	2099.7	23093.9	1780.9	0.093	0.187
Depressive disorder	5427.6	630.5	5929.5	585.5	0.014 *	0.709
Bipolar disorder	929.0	91.7	1054.8	44.4	<0.001 *	0.263
Primary insomnia	281.3	39.5	271.7	32.3	0.379	0.763
Schizophrenia	594.3	24.1	503.8	13.9	<0.001 *	0.014 †
Panic disorder	469.8	58.6	643.7	78.8	<0.001 *	0.113
Hypochondriasis	10.0	3.4	14.1	3.4	0.001 *	0.871
Post traumatic stress disorder	158.8	17.1	170.3	17.8	0.066	0.762
Anxiety disorder	2663.4	255.7	3186.3	411.4	<0.001 *	0.542
Anorexia nervosa	13.0	2.9	12.2	3.0	0.295	0.611
Adephagia	12.6	5.0	11.0	3.0	0.497	0.443
Women						
Autism	474.8	44.6	589.9	71.8	<0.001 *	0.353
ADHD	4617.1	507.0	5573.9	562.3	<0.001 *	0.425
Depressive disorder	8845.5	1416.1	9819.5	1074.3	0.021 *	0.973
Bipolar disorder	1067.0	148.0	1361.9	98.2	<0.001 *	0.101
Primary insomnia	294.2	44.3	312.2	21.9	0.159	0.003 †
Schizophrenia	572.1	27.9	485.3	10.7	<0.001 *	0.017 †
Panic disorder	588.2	100.7	863.4	137.4	<0.001 *	0.165
Hypochondriasis	5.6	2.4	12.2	8.1	0.006 *	0.727
Post traumatic stress disorder	257.4	28.5	295.3	29.0	<0.001 *	0.777
Anxiety disorder	2873.2	422.4	3716.3	585.5	<0.001 *	0.898
Anorexia nervosa	93.4	10.0	114.9	23.4	0.003 *	0.081
Adephagia	66.5	9.6	69.6	8.1	0.464	0.828

* Mann–Whitney U test, significance at <0.05, † Levene’s test in non-parametric data, significance at <0.05.

**Table 3:** Mean, Standard deviation of incidence of diseases before and during COVID-19, and their difference in the subgroup by age.

Diseases	Before COVID-19		During COVID-19		*p*-Values of Difference	
	Mean	SD	Mean	SD	Mean	Variance
Age 0–4 years old						
Autism	300.3	29.7	384.9	74.1	<0.001 *	0.526
ADHD	94.8	16.4	118.9	35.9	0.026 *	0.380
Depressive disorder	14.2	4.7	8.5	2.5	<0.001 *	0.140
Bipolar disorder	2.1	1.3	2.3	1.3	0.592	0.651
Primary insomnia	12.6	3.9	14.5	5.1	0.187	0.562
Schizophrenia	0.6	0.6	0.4	0.5	0.433	0.142
Panic disorder	0.3	0.5	0.5	0.6	0.130	0.036 †
Hypochondriasis	0.5	0.9	0.5	0.7	0.715	0.539
Post traumatic stress disorder	9.2	3.0	7.7	3.5	0.196	0.277
Anxiety disorder	52.8	9.1	62.1	16.9	0.136	0.432
Anorexia nervosa	2.7	2.1	3.1	1.9	0.473	0.803
Adephagia	4.3	4.3	3.9	1.7	0.663	<0.001 †
Age 5–9 years old						
Autism	1141.3	143.2	1421.1	180.5	<0.001 *	0.560
ADHD	9660.0	1334.1	10395.3	915.3	0.074	0.010 †
Depressive disorder	324.0	43.3	367.2	47.2	0.007 *	0.343
Bipolar disorder	45.7	9.1	60.3	3.8	<0.001 *	0.135
Primary insomnia	9.6	3.7	12.4	4.4	0.009 *	0.178
Schizophrenia	8.8	2.0	9.1	3.0	0.891	0.160
Panic disorder	7.3	2.6	6.8	1.5	0.564	0.102
Hypochondriasis	0.5	0.7	1.1	1.0	0.074	0.122
Post traumatic stress disorder	41.8	5.7	42.3	7.1	0.989	0.360
Anxiety disorder	372.1	32.7	454.5	70.0	<0.001 *	0.422
Anorexia nervosa	3.2	2.2	5.5	2.3	0.003 *	0.956
Adephagia	3.3	2.9	2.5	1.6	0.618	0.594
Age 10–14 years old						
Autism	752.3	87.2	974.3	104.1	<0.001 *	0.145
ADHD	10797.7	916.1	11839.2	1088.5	0.004 *	0.978
Depressive disorder	2550.7	454.4	2366.9	338.9	0.110	0.023 †
Bipolar disorder	272.1	39.0	294.3	28.7	0.113	0.047 †
Primary insomnia	37.5	4.6	40.1	7.4	0.316	0.195
Schizophrenia	80.8	8.0	86.9	10.7	0.042 *	0.019 †
Panic disorder	84.2	13.7	122.1	31.4	0.001 *	0.368
Hypochondriasis	2.2	1.0	5.1	3.0	0.001 *	0.200
Post traumatic stress disorder	98.9	18.4	109.9	17.8	0.070	0.608
Anxiety disorder	920.1	110.7	1133.1	216.6	0.001 *	0.206
Anorexia nervosa	41.8	6.1	49.9	13.0	0.030 *	0.023 †
Adephagia	7.0	1.9	8.3	3.8	0.557	0.055
Age 15–19 years old						
Autism	876.7	33.9	770.3	25.8	<0.001 *	0.018 †
ADHD	5945.7	388.1	6314.5	375.9	0.003 *	0.776
Depressive disorder	11384.3	1574.8	13006.3	1292.9	0.002 *	0.861
Bipolar disorder	1676.0	197.1	2059.7	110.6	<0.001 *	0.094
Primary insomnia	515.8	77.2	517.0	43.2	0.935	0.010 †
Schizophrenia	1076.3	43.4	892.7	26.2	<0.001 *	0.014 †
Panic disorder	966.2	146.9	1377.7	184.0	<0.001 *	0.127
Hypochondriasis	12.4	4.3	19.7	6.9	0.001 *	0.417
Post traumatic stress disorder	266.3	25.4	305.7	23.0	<0.001 *	0.612
Anxiety disorder	4191.5	541.5	5252.9	716.8	<0.001 *	0.975
Anorexia nervosa	58.7	5.8	68.6	10.7	0.001 *	0.168
Adephagia	64.5	10.2	65.5	6.4	0.818	0.087

* Mann–Whitney U test, significance at <0.05, † Levene’s test in non-parametric data, significance at <0.05.

## Data Availability

Releasing of the data by the researcher is not legally permitted. All data are available from the database of the Korea Center for Disease Control and Prevention. The Korea Center for Disease Control and Prevention allows data access, at a particular cost, for any researcher who promises to follow the research ethics. The data of this article can be downloaded from the website after agreeing to follow the research ethics.
